# DNA Methylation Biomarkers in Aging and Age-Related Diseases

**DOI:** 10.3389/fgene.2020.00171

**Published:** 2020-03-10

**Authors:** Yasmeen Salameh, Yosra Bejaoui, Nady El Hajj

**Affiliations:** College of Health and Life Sciences, Hamad Bin Khalifa University, Doha, Qatar

**Keywords:** aging, DNA methylation, epigenetic clocks, biomarkers, Alzheimer’s disease, diabetes, cardiovascular diseases

## Abstract

Recent research efforts provided compelling evidence of genome-wide DNA methylation alterations in aging and age-related disease. It is currently well established that DNA methylation biomarkers can determine biological age of any tissue across the entire human lifespan, even during development. There is growing evidence suggesting epigenetic age acceleration to be strongly linked to common diseases or occurring in response to various environmental factors. DNA methylation based clocks are proposed as biomarkers of early disease risk as well as predictors of life expectancy and mortality. In this review, we will summarize key advances in epigenetic clocks and their potential application in precision health. We will also provide an overview of progresses in epigenetic biomarker discovery in Alzheimer’s, type 2 diabetes, and cardiovascular disease. Furthermore, we will highlight the importance of prospective study designs to identify and confirm epigenetic biomarkers of disease.

## Introduction

Aging is a complex and time-dependent deterioration of physiological process occurring in the majority of living organisms (Galloway, 1993). In humans, life expectancy has increased rapidly in the last few centuries due to a significant improvement in medical care and public health awareness (Crimmins, 2015). Consequently, increased life expectancy caused higher morbidity rates since advanced age is a predominant risk factor for several diseases including cancer, dementia, diabetes, and cardiovascular disease (CVD) (Jaul and Barron, 2017; Franceschi et al., 2018). Currently, there is an urgent need to improve health and longevity to increase not just the life span but also the health span of the elderly population. In recent years, several molecular and cellular processes have been reported to be linked to aging and contribute to its phenotype. Scientists proposed nine hallmarks of aging that can be classified into three categories: primary, antagonistic, or integrative (López-Otín et al., 2013). The primary hallmarks are defined as key factors causing cellular damage including genomic instability, telomere attrition, loss of proteostasis, and epigenetic alterations (López-Otín et al., 2013). During aging, there is a continuous accumulation of epigenetic changes, which might give rise to multiple age-related pathologies. A number of epidemiological studies revealed that monozygotic twins exhibit an increased rate of phenotypic discordance particularly for age-related diseases among older siblings (Frederiksen et al., 2002; Reynolds et al., 2005; Zwijnenburg et al., 2010; Greenwood et al., 2011; Castillo-Fernandez et al., 2014). This may be due to a gradual decrease in methylation conservation rates with successive cell divisions, a phenomenon referred to as “Epigenetic Drift” (Poulsen et al., 2007; Issa, 2014). This notion proposes an increased rate of stochastic methylation errors across the entire genome during aging. Indeed, several reports provided compelling evidence that older monozygotic twins exhibit global differences in DNA methylation (DNAm) patterns when compared to their younger counterparts (Fraga et al., 2005; Lévesque et al., 2014; Tan et al., 2016; Wang et al., 2018). Similarly, a centenarian’s methylome displays reduced DNA methylation levels as well as a decreased pair-wise correlation in the methylation status of neighboring CpG sites relative to the methylome of a newborn (Heyn et al., 2012).

In 1973, Vanyushin et al. (1973) were the first to describe global 5-methylcytosine (5mC) variations during aging in rats. Now, vastliterature have revealed genome-wide DNA methylation changes that occur in response to aging across multiple species. These age-related epigenetic alterations either arise systemically or are restricted to a specific tissue/cell type. Age-related DNA methylation changes also take place in germ cells and might be possibly transmitted to the offspring (Atsem et al., 2016; Potabattula et al., 2018). Since the sequencing of the human genome the scientific community has been trying to elucidate how the genetic code controls the spatial and temporal expression of genes. The essence of DNA lies within the dynamic interaction between the genetic sequence (i.e. genome) and the epigenome. In many ways, environmental influences alter gene expression through various mechanisms such as DNA methylation, hydroxymethylation, histone modifications, alternative splicing, etc. (Edwards and Myers, 2007). Recent advances in “omics” technologies availed new avenues toward implementing precision medicine based on the genetic, environmental, and lifestyle factors of each individual. Similarly, treatments of complex diseases is demanding better diagnostic and screening tools for early detection particularly in the initial phase of the disease. DNA methylation (5-methylcytosine) is a covalent epigenetic modification to the DNA by addition of a methyl group to the C-5 position of the cytosine ring by DNA methyltransferases (Dnmts). Whereas, DNA hydroxymethylation (5-hydroxymethylcytosine) is a more recently discovered modification involving the addition of a hydroxymethyl group to the 5′ position of cytosine. DNA hydroxymethylation has been reported to be enriched in the brain especially in the proximity of synaptic genes (Kriaucionis and Heintz, 2009; Khare et al., 2012). The role 5-hmC plays in various biological processes remains elusive, nevertheless scientists are starting to appreciate its importance in gene expression regulation. Methylation and demethylation processes are not only important for transcription regulation but also play a crucial role during development and cell differentiation (Moore et al., 2013). Recently, DNA methylation measurements were shown to be valuable age prediction tools, even surpassing in accuracy the age prediction models based on telomere length (Horvath et al., 2016a). DNA methylation-based age prediction models are not only accurate in predicting chronological age but can also estimate biological aging rates (Chen et al., 2016; Christiansen et al., 2016).

## Epigenetic-Based Aging Clocks

**FIGURE 1:**
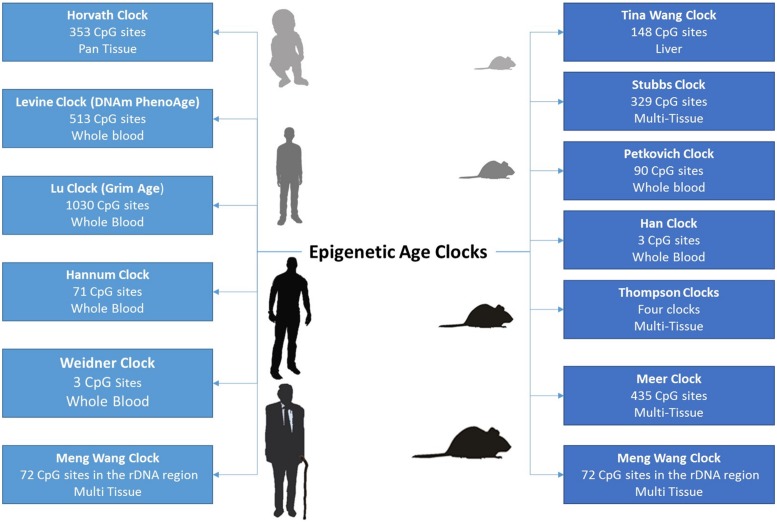
The growing number of epigenetic age clocks developed for both humans and mice, including the number of CpG sites comprising the age-prediction model, as well as the tissues in which age can be estimated.

It is only 6 years since Steve Horvath inaugurated a new era in epigenetics and aging research. In a landmark study, he developed a multivariate age predictor based on DNA methylation values of 353 individual CpG sites (Horvath, 2013). One of the main advantages of the Horvath clock is its ability to predict age systemically in all human cell types and tissues, excluding sperm. This is in contrast to other clocks that can be only applied to a single tissue (Hannum et al., 2013; Figure 1). Interestingly, the clock starts ticking early during development where fetal tissues as well as embryonic and induced pluripotent stem cells reveal a DNA methylation age (DNAm age) between −1 and 0 years (Horvath, 2013; Spiers et al., 2015). Till now, the biological mechanisms underlying changes measured by the epigenetic age clock have not been clearly identified. Therefore, recognizing genes that influence the rate of epigenetic aging might help determine such biological processes. Recent genome-wide association studies revealed tissue-specific association of variants in metabolism, immune system, aging, and autophagy -related genes with epigenetic age acceleration (Kananen et al., 2016; Lu et al., 2016, 2017, 2018). Epigenetic clocks have been also proposed to measure molecular processes involved in development and tissue homeostasis particularly those affecting stem cell differentiation as well as replenishment of committed cells (Horvath and Raj, 2018).

**FIGURE 2:**
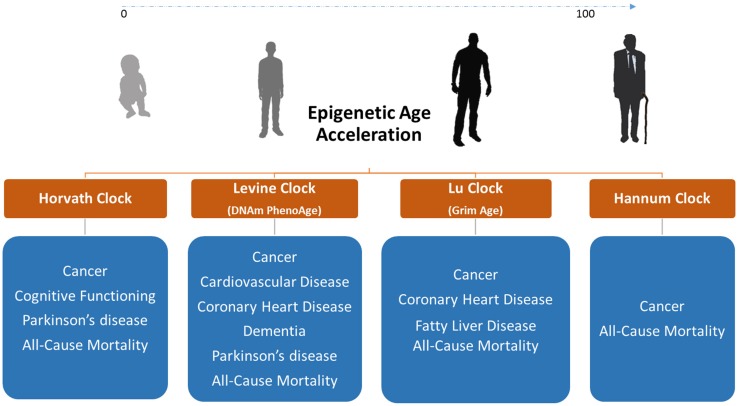
Diseases and conditions associated with DNAm age acceleration in blood DNA where epigenetic clocks can be used as biomarkers of disease. We only display diseases/conditions where epigenetic age acceleration is observed in blood or other non-invasive tissues. We do not show correlations with glucose, insulin, HDL, and triglyceride levels as well as with blood pressure since these factors are biomarkers on their own.

By regressing DNAm age on chronological age, epigenetic clocks can determine whether biological age acceleration occurs in certain diseases or in response to environmental factors (Horvath and Raj, 2018). Using this approach, age acceleration measurements in blood were associated with body mass index (BMI), obesity, physical fitness, Huntington’s disease, Parkinson’s disease, sleep, and smoking (Horvath et al., 2014; Horvath and Ritz, 2015; Horvath et al., 2016b; Carroll et al., 2017; Quach et al., 2017; Levine et al., 2018). Epigenetic clocks are highly valuable age prediction tools nevertheless their true value as diagnostic biomarkers requires further confirmation (Figure 2). Such biomarkers are epigenetic modifications/marks used as a risk assessment and diagnostic tool to uncover sequence of events preceding the manifestation of disease. Biomarkers can be measured within tissue or body fluid, in the context of disease vs health state, for the purpose of disease detection, disease prognosis, response to therapy, and therapy monitoring (García-Giménez et al., 2016).

Evidently, epigenetic clocks were employed to study epigenetic age acceleration in age-related disorders. For e.g. several reports showed DNAm age acceleration associated with incidence, future onset, and mortality across several types of cancer (Levine et al., 2015a; Zheng et al., 2016; Ambatipudi et al., 2017). Similarly, DNAm age was reported to be a useful biomarker for predicting physical and mental fitness in elderly individuals (Marioni et al., 2015) and was shown to be associated with cholesterol (High Density Lipoprotein: HDL), insulin, glucose, and triglycerides levels (Quach et al., 2017; Levine et al., 2018). The adult progeroid disease, Werner syndrome, which mimics aging at a faster rate, also revealed DNAm age acceleration of >6 years (Maierhofer et al., 2017). Recently, the Horvath lab developed the DNAm PhenoAge clock by training their predictor on phenotypic age rather than chronological age (Levine et al., 2018). The DNAm PhenoAge is a powerful biomarker for measuring health- and life- span that relies on measurements from 513 CpG sites (Levine et al., 2018). This clock could conclusively predict CVD incidence using whole blood DNA methylation values. In 2019, the DNAm GrimAge clock was released where it was reported to predict mortality, cancer, and coronary heart disease (CHD) to a high level of accuracy (Lu et al., 2019). Epigenetic clocks that can estimate gestational age of neonates are also available (Knight et al., 2016). Using these clocks, we have demonstrated that DNAm age of children born via intracytoplasmic sperm injection (ICSI) lags half a week behind their naturally conceived counterparts (El Hajj et al., 2017).

In mice, epigenetic aging clocks were recently developed by relying on reduced representation bisulfite sequencing (RRBS) or whole genome bisulfite sequencing (WGBS) data (BI Ageing Clock Team et al., 2017; Petkovich et al., 2017; Wang et al., 2017; Meer et al., 2018; Thompson et al., 2018). These clocks provide useful biomarkers for measuring whether experimental interventions are able to slow the aging process in mice. Current research is focused on identifying evolutionary conserved pan-mammalian clocks that can calculate age across multiple species with varying lifespans. In addition, efforts are being invested in identifying clocks based on a handful of CpG sites since methylation arrays, RRBS, or WGBS remain relatively expensive compared to bisulfite pyrosequencing. In this aspect, Wolfgang Wagner’s group has shown that measurements from just three CpG sites can accurately readout lifespan in both humans and mice (Weidner et al., 2014; Han et al., 2018). More recently, an epigenetic clock based on ribosomal DNA methylation was reported to be evolutionary conserved across several species (Wang and Lemos, 2019).

## Epigenetic Dysregulation in Type 2 Diabetes, Alzheimer’s Disease, and Cardiovascular Disease

The dynamic change between methylation and demethylation states introduces flexibility to the rigidly stable DNA code, allowing controlled changes in gene expression in response to external and internal environmental cues. These moldable, yet generally stable processes are becoming valuable tools for distinguishing healthy versus diseased states. In cancer, despite the genome-wide hypomethylation, CpG islands are hypermethylated and can serve as a biomarker for early cancer detection (Anglim et al., 2008). Recent studies have shown that changes in global content of 5mC and 5hmC are not only useful as early detection tools but also a valuable source for understanding the underlying mechanisms of cancer development and patient prognosis (Liu et al., 2019). There are several published reviews discussing epigenetic biomarkers in cancer, however, this review’s main focus will be on DNA methylation biomarkers in type 2 diabetes (T2D), Alzheimer’s disease (AD), and cardiovascular disease (CVD) (Supplementary Table S1). Here, it is important to mention that these biomarkers are independent of the epigenetic clock described in the previous section.

### Type 2 Diabetes

According to the World Health Organization (WHO), >420 million adults suffer from diabetes where 1.6 million deaths per year are directly attributed to the disease (Chan, 2014). The increased lifespan in humans is one of the main contributors to the rising prevalence of diabetes in the older population. Currently, more than third the United States population above the age of 65 are diabetics with numbers projected to increase in the next decade. Type 2 Diabetes (T2D) is a metabolic disorder characterized by abnormally elevated blood glucose levels due to β-cells dysfunction and insulin resistance (Chatterjee et al., 2017). T2D is a complex multifactorial disease where a variety of genetic, epigenetic, and environmental factors contribute to its etiology (McCarthy, 2010). Common complications of diabetes include cardiovascular problems, neuropathy, nephropathy, and retinopathy due to high blood glucose levels (Jacobs et al., 2017). Therefore, prevention or early treatment are very important to prevent damage to several of the body’s systems. Despite the availability of well-established measures for diagnosing diabetes such as hemoglobin A1c (HbA1c) and fasting glucose, additional DNA-methylation based biomarkers can help complement current tests for screening and diagnosis. Identifying an individual during the pre-diabetic stage is very important for the management of the disease since ∼70% of persons with intermediate hyperglycaemia tend to develop T2D later in life.

Recently, efforts have focused on defining epigenetic risk factors associated with T2D as well as its major risk factors. Published reports have identified DNAm alterations in various tissues of T2D patients including blood, liver, pancreas, skeletal muscle, and adipose tissue (Ling and Rönn, 2019). These studies employed different approaches to quantify methylation changes including candidate gene analysis, global 5mC measurements, DNA methylation arrays, as well as WGBS (Volkov et al., 2017; Ling and Rönn, 2019). Evidently, the first reports describing epigenetic dyrsegulation in skeletal muscle and pancreatic islets of T2D patients applied a candidate gene approach. These studies identified increased DNA methylation and reduced gene expression in T2D-related genes such as *INS*, *PDX1*, *PPARGC1A*, and *GLP1R* (Ling et al., 2008; Barrès et al., 2009; Yang et al., 2012; Hall et al., 2013). Similarly, bisulfite pyrosequencing and methylation-specific PCR were employed to study methylation of key T2D genes in blood DNA. Investigated genes included *KCNJ11, PPARgamma, PDK4, KCNQ1, PDX1, FTO, PEG3, TCF7L2, GCK, PRKCZ, BCL11A,GIPR, SLC30A8, IGFBP-7, PTPPN1, CAMK1D, CRY2, CALM2, TLR2, TLR4*, and *FFAR3* [reviewed in Willmer et al. (2018)]. Most of those studies suffered from low sample size apart of a report by Seman et al. (2015), which quantified methylation in the solute carrier family 30 member 8 *(SLC30A8)*. Here, the authors detected hypermethylation at several CpG sites in *SLC30A8* in 516 T2D subjects vs 476 individuals with normal glucose tolerance (Seman et al., 2015). Global changes in DNAm levels were also investigated using bisulfite pyrosequencing of *ALU* and *LINE-1* elements, liquid chromatography mass spectrometry, Imprint Methylated DNA Quantification kit (Sigma-Aldrich), and High Performance Liquid Chromatography (HPLC). Conflicting results were reported which might be inherently related to low sample size and lack of replication in independent cohorts [reviewed in Willmer et al. (2018)].

The development of Infinium Methylation arrays and NGS-based methylation sequencing allowed simultaneous quantification of methylation at thousands of CpG sites. Several case-control array studies compared DNA methylation abnormalities in pancreatic islets, liver, and subcutaneous adipose tissue of T2D patients. The focus of this review is on methylation-based biomarkers therefore we will mainly describe changes reported in blood or other accessible tissues. One impressive example of such alterations is the occurrence of dynamic DNA methylation changes in Peripheral Blood Mononuclear Cells (PBMCs) ∼80–90 days prior to elevated glucose levels. This was observed by Chen et al. (2018) after longitudinally following a healthy individual over the course of 3 years while measuring DNA methylation levels using WGBS at 28 selected time-points. Another study by Toperoff et al. (2012) used a pooling-based methylation screen followed by individual-level replication in a prospective cohort to identify CpGs that can predict future T2D risk. The authors reported a single CpG site in the first intron of the fat mass and obesity-associated (FTO) gene to be hypomethylated prior to the appearance of T2D (Toperoff et al., 2012). DNA methylation alterations were also measured in concordant and discordant monozygotic twins for T2D using genome-wide methylated DNA immunoprecipitation sequencing (MeDIP-seq). This elegantly designed study uncovered differentially methylated regions (DMRs) located in the promoters of *MALT1* and *GPR61* (Yuan et al., 2014).

In addition to age, BMI is a major risk factor contributing to T2D and has been the focus of multiple epigenome-wide association studies (EWAS) studies. A large study on >10,000 samples identified DNA methylation changes across 187 loci correlating with high BMI levels. Out of the 187 “sentinel obesity biomarkers,” 62 loci were associated with T2D incidence including a probe in *ABCG1* with the strongest significance. A methylation risk score based on the sum of these markers exhibited a higher predictive power of future T2D onset when compared to traditional risk factors such as obesity, fasting glucose, and hyperinsulinemia (Wahl et al., 2017). Similarly, a longitudinal follow-up study on Indian Asians and Europeans discovered five T2D methylation markers in whole blood DNA collected at baseline prior to diabetes onset. These markers located in *ABCG1*, *PHOSPHO1*, *SOCS3*, *SREBF1*, and *TXNIP* were associated with metabolic measures of insulin resistance including glucose concentration, BMI, waist-to-hip ratio, and homeostatic model assessment for insulin resistance (HOMA-IR) (Chambers et al., 2015). A conceptually related study tried to replicate the association between T2D and the five previously mentioned genes in subjects from the Botnia prospective cohort. Nonetheless, they could only confirm *ABCG1* and *PHOSPHO1* methylation as predictors of future T2D risk (Dayeh et al., 2016). This association was also observed in healthy individuals where *ABCG1* methylation was reported to correlate with fasting insulin and HOMA-IR (Hidalgo et al., 2014).

Further EWAS studies could confirm methylation aberrations in some of the previously mentioned genes. A large EWAS analysis in Mexican-American individuals unraveled five CpG sites linked to T2D-related traits out of which 3 were located in *TXNIP (cg19693031)*, *ABCG1*, and *SAMD12* (Kulkarni et al., 2015). Two separate studies from Spain and Germany confirmed the association between decreasing methylation levels at *TXNIP (cg19693031)* and T2D, as well as with fasting glucose and HbA1c concentrations (Florath et al., 2016; Soriano-Tárraga et al., 2016). To end with EWAS, it is important to mention a meta-analysis by Walaszczyk et al. (2018) that took the initiative to confirm potential glycemic trait and T2D biomarkers. In this replication analyses, the authors concluded that a significant association between T2D and methylation sites in *ABCG1*, *TXNIP*, and *SREBF1* exists, which makes them promising biomarkers for early T2D detection. As a final point, we have to emphasize the significance of non-genetic elements including blood sugar levels, patient age, BMI, and gender in predicting future diabetes risk. Thus, such factors should be integrated into a T2D predictive model that includes genetic and epigenetic biomarkers to improve early T2D detection and allow better disease prognosis.

### Alzheimer’s Disease

Accumulation of errors in the epigenetic machinery during aging progression increases the risk for onset of age-related pathologies, such of those involving brain deterioration and neurodegeneration. The most common brain disorders affecting elderly individuals are those causing dementia through loss of synaptic plasticity, leading to memory impairment and defective learning capabilities. Alzheimer’s disease (AD) affects 45–60% of the population with dementia and its burden is expected to double by the year 2060 (Finder, 2010; Duong et al., 2017). AD is a polygenic, complex and age-related neurodegenerative disease clinically characterized by progressive memory loss and cognitive impairment. Its pathological features include accumulation of β-amyloid (Aβ) in senile plaques, the formation of neurofibrillary tangles (NFTs) composed of hyperphosphorylated protein tau, and massive neuronal loss mainly in the hippocampus as well as associated regions of the neocortex (Hardy, 2006). Several clinical and epidemiological aspects of AD indicate a role for epigenetic factors in its etiology. This is evident in monozygotic twins discordant for the disease where prognosis and age-of onset could vary by >10 years. Indeed, a broad spectrum of epigenetic pathways such as DNA methylation, histone modification, and non-coding RNAs (ncRNAs) appear to be aberrant. For e.g. Wang et al. (2008) reported that Alzheimer’s susceptibility loci have an age-specific epigenetic drift in brain and blood of individuals with late-onset AD. Several studies were conducted to identify epigenetic aberrations, as well as to differentiate specific methylation changes occurring in AD vs non-AD dementias [reviewed in: Lardenoije et al. (2015)]. Using southern blot analysis, West et al. (1995) first showed loss of methylation at a single site in the amyloid precursor protein (APP) gene in postmortem human brain of a single individual with AD. This was confirmed by Tohgi et al. (1999) who reported that hypomethylation of cytosine residues within the APP promoter with age results in Aβ deposition in the cerebral cortex of human autopsy brain samples. Nevertheless, new studies using bisulfite sequencing failed to replicate these findings (Brohede et al., 2010). Recently, neuronal fractions from postmortem brains of Alzheimer’s patients were reported to display significantly up-regulated expression of *BRCA1*, consistent with hypomethylation of a CpG island (CGI) in its promoter region. BRCA1 protein levels were also increased in response to Aβ deposition and became mislocalized to the cytoplasm, in both *in vitro* cellular and *in vivo* mouse models (Mano et al., 2017).

After the introduction of methylation arrays, a large study on >700 autopsied brain samples revealed methylation and expression changes in *ANK1*, *CDH23*, *DIP2A*, *RHBDF2*, *RPL13*, *SERPINF1*, and *SERPINF2* (De Jager et al., 2014). Similarly, Lunnon and collaborators performed a large EWAS analysis on four brain regions where they reported a significant hypermethylation of *ANK1* in the entorhinal cortex, superior temporal gyrus, and prefrontal cortex of AD individuals. The authors went on to measure methylation in pre-mortem blood DNA where they identified distinct differentially methylated probes (DMPs) to those in AD brains (Lunnon et al., 2014). The top ranked AD-associated blood DMPs were located in *DAPK1*, *GAS1*, and *NDUFS5*. Furthermore, epigenetic age acceleration was shown to be associated with AD neuropathological markers such as neuritic plaques, diffuse plaques, and amyloid load in the dorsolateral prefrontal cortex (Levine et al., 2015b). Down’s syndrome patients, predisposed to early onset AD, also display DNAm age acceleration in blood and brain tissue starting early during *in utero* development (Horvath et al., 2015; El Hajj et al., 2016) in addition to epigenetic dysregulation at the clustered protocadherin locus (Almenar-Queralt et al., 2019).

Presently, a definitive AD diagnosis is only possible through neuropathological examination of brain tissue after death. Therefore, it is important to identify clinical biomarkers that can help in early disease detection. In addition, the effectiveness of available FDA-approved treatments for AD increases when administered during early stages of the disease. Currently, ongoing research efforts are mainly focused on delineating AD-related epigenetic changes that occur in various brain regions. So far, only a limited number of studies have assessed DNA methylation changes in blood cells. These articles will be the subject of the next section, where we will first summarize findings observed using a candidate gene approach. In one of these studies, blood DNA methylation of the Brain-derived neurotrophic factor gene (*BDNF*) promoter and a tag SNP (rs6265) were shown to have a significant role in the progression of the amnestic mild cognitive impairment (aMCI) to AD. Here, the interaction between DNA methylation of CpG5 and AA genotype of rs6265 had a role in the progression of aMCI to AD (*p* = 0.003, OR = 1.399, 95% CI: 1.198–1.477) (Xie et al., 2017a). A 5-year longitudinal study also revealed *BDNF* promoter methylation as a significant independent predictor of aMCI to AD transformation (Xie et al., 2017b). Similarly, Nagata et al. (2015) reported higher DNA methylation affecting a single CpG site in the *BDNF* promoter of patients with AD. Nevertheless, it is important to note that Carboni et al. (2015) could not confirm methylation alterations in the *BDNF* promoter in peripheral blood of Alzheimer’s disease patients. Therefore, doubts remain as to whether *BDNF* promoter methylation changes occur in AD patients. Besides, DNA methylation levels were demonstrated to be significantly elevated in Coenzyme A Synthase (*COASY*) and Serine Peptidase Inhibitor (*SPINT1*) gene promoter regions in AD and aMCI (Kobayashi et al., 2016). DNA methylation at the *NCAPH2/LMF2* promoter region was also found to be a useful biomarker for the diagnosis of AD and aMCI where it was shown to be associated with hippocampal atrophy through apoptosis (Shinagawa et al., 2016). Furthermore, Ozaki et al. (2017) could show that a decline in DNA methylation in intron 1 of Triggering receptor expressed on myeloid cells 2 gene (*TREM2*) causes higher mRNA expression in the leukocytes of AD subjects versus controls. Phosphatidylinositol Binding Clathrin Assembly Protein (*PICALM*) was another candidate gene whose methylation associated with cognitive decline in blood cells of AD patients (Mercorio et al., 2018). Higher global DNA methylation levels were also observed in the peripheral blood mononuclear cells of late onset Alzheimer disease (LOAD) patients. This hypermethylation was associated with APOEε4 allele (*p* = 0.0043) and APOEε3 carriers (*p* = 0.05) (Di Francesco et al., 2015). In the same way, Bollati et al. (2011) observed a hypermethylation of *LINE-1* elements in AD patients after measuring DNA methylation at *ALU*, *LINE-1*, and alpha satellite repetitive elements.

In AD, epigenome-wide association studies (EWAS) on prospective cohorts are still lacking. To address this limitation, the German Study on Aging, Cognition and Dementia in Primary Care Patients (AgeCoDe) recruited >3300 healthy individuals at baseline to investigate markers for early detection of dementia and cognitive impairment. From this cohort, Lardenoije et al. (2019) identified 55 converters healthy at baseline that developed AD dementia at follow-up. Using DNA methylation arrays, several differentially methylated regions were spotted in blood of AD converters at baseline. By focusing on those regions, we could discern epigenetic dysregulation at six DMPs in blood DNA of Down’s syndrome patients who are at high risk of developing early onset AD. One of the DMPs mapped to *ADAM10*, a major alpha-secretase, responsible for APP cleavage in neurons (Haertle et al., 2019). It is still challenging to find a non-invasive biomarker that reflects AD pathogenesis in the brain. Nonetheless, the previously described epigenetic alterations might be considered potential biomarkers that require further research to assess their efficacy.

### Cardiovascular Disease

Cardiovascular disease (CVD) is an umbrella term for a range of conditions that affect the heart or blood vessels. The main determinants of a person’s cardiovascular health is age, as well as several risk factors including diabetes, smoking, obesity, and high blood pressure. Epigenetic aging biomarkers based on “The Horvath Clock,” “DNAm PhenoAge,” and “DNAm GrimAge” were recently reported to be associated with CVD risk (Levine et al., 2018; Lind et al., 2018; Lu et al., 2019). Even though not much research is published on the epigenetics of CVD, however, the impact of epigenetics has been extensively studied in the aforementioned risk factors. The complex interplay of genetics, epigenetics, and environment have an important role in the pathogenesis and progress of these conditions. For e.g. a *trans-*ancestry genome wide association study (GWAS) identified 12 genetic variants associated with methylation levels, which influences susceptibility for hypertension (Kato et al., 2015). Similarly, elevated global DNA methylation levels were reported to be positively associated with CVD and its predisposing risk factors (Sharma et al., 2008; Kim et al., 2010). Another example by Infante et al. (2019) investigated DNA methylation and expression changes in coronary heart disease patients undergoing Cardiac Computed Tomography (CCT). They could show that genes involved in cholesterol bioactivity such as *LDLR* promoter have higher methylation in PBMNCs of CHD patients compared to healthy controls. *LDLR* promoter methylation was also associated with calcified plaque volume and total plague burden measured via CCT. A case-control study using Human CpG 12K Array (HCGI12K) revealed 72 DMRs hyper-methylated in patients with coronary artery disease (CAD) (Sharma et al., 2014). More recently, an EWAS analyses using the HumanMethylation450 BeadChips reported 211 CpG sites located on 196 genes to be differentially methylated in patients with a history of myocardial infarction (MI) (Rask-Andersen et al., 2016). A similar EWAS study on acute coronary syndrome revealed associations with blood methylation levels of 47 CpG sites located in genes involved in atherogenic signaling and immune response (Li et al., 2017). Nakatochi et al. (2017) also performed an EWAS analyses on blood DNA of patients suffering from MI which revealed three differentially methylated CpG sites in *SGK1, SMARC4*, and *ZFHX3*. A large EWAS study on the Women’s Health Initiative (discovery set) and Framingham Heart Study (FHS) – (replication set) identified three DMRs in *SLC9A1*, *SLC1A5*, and *TNRC6C* linked to CVD incidence (Westerman et al., 2018). The authors also performed a module based epigenetic analysis, which revealed three modules associated with CVD and its risk factors out of which two had strong concordance in both cohorts (Westerman et al., 2018).

A growing number of studies reported a possible role for DNA methylation in atherosclerosis pathogenesis (Newman, 1999; Napoli et al., 2012; Aavik et al., 2015; Liu et al., 2018). Atherosclerotic lesions are known to harbor differentially methylated CpGs in genes involved in endothelial and smooth muscle functions (Zaina et al., 2014). Circulating concentrations of tumor necrosis factor α, a pro-inflammatory cytokine linked to atherosclerosis, were recently shown to be associated with methylation changes in the immune response-related genes *DTX3L-PARP9* and *NLRC5.* DNA methylation levels of those genes were also shown to negatively correlate with CHD incidence (Aslibekyan et al., 2018). Similarly, a large EWAS meta-analysis on serum C-reactive protein (CRP), an inflammation biomarker predicting heart failure, identified 58 CpG sites related to CRP levels. Several of those CpGs (51 sites) were associated with cardio-metabolic traits including CHD prevalence and incidence (Ligthart et al., 2016). More recently, focus shifted toward understanding the role of 5-Hydroxymethylcytosine in CVD, where reports have shown that global DNA hydroxymethylation levels could be better predictors of MI and CHD when compared to 5-mC. In elderly individuals, the incidence and degree of coronary atherosclerosis (CA) were linked to increased DNA hydroxymethylation levels in PBMCs (Jiang et al., 2019a). This lead the authors to propose a novel CA biomarker based on integrating carotid plaques scores, as well as DNA methylation and hydroxymethylation data (Jiang et al., 2019b).

From a precision health perspective, a machine learning based framework focused on the FHS cohort could detect CHD presence and foresee its incidence by implementing genetic, epigenetic and phenotypic data (Dogan et al., 2018a, b). Similarly, DNA methylation levels in the *TRAF3* gene were reported to predict recurrence of ischemic events in patients treated with Clopidogrel (Gallego-Fabrega et al., 2016b). A conceptually related study from the same group identified *PPM1A* methylation to be associated with vascular recurrence after stroke in aspirin treated patients (Gallego-Fabrega et al., 2016a). Nonetheless, there must be a more concerted effort to establish whether the reported epigenetic alterations can be reliable CVD biomarkers.

## Conclusion and Future Perspectives

Despite the extensive plethora of epigenetic modifications, measuring DNA methylation of specific CpG sites remains the most promising epigenetic biomarker. DNA methylation modifications are highly stable compared to RNA- or protein-based biomarkers, relatively easy to measure using non-invasive biospecimen, and are quantifiable marks on the DNA that can track the influences of various environmental and lifestyle factors (Berdasco and Esteller, 2019). Nevertheless, epigenetic biomarkers are still in the nascent stage and more research is warranted to move toward applications in healthcare. Still, efforts invested in developing biomarkers based on the epigenetic clocks has accelerated discoveries in the field. Furthermore, GRAIL a multi-billion dollar investment has chosen DNA methylation as its preferred approach for a non-invasive test for early cancer detection.

A key factor in the development of epigenetic clocks was the advent of Infinium Methylation arrays that enabled simultaneous quantification of DNA methylation starting from ∼27,000 individual CpG sites (Infinium HumanMethylation27 BeadChip) up to 850,000 sites via EPIC arrays. These methylation arrays provide a cost-effective approach for large-scale epigenetic epidemiology studies. Nevertheless, the human genome is comprised of 28 million CpG sites out of which 3% are measured using Epic Arrays. Even though, a few reports have mentioned that whole genome bisulfite sequencing (WGBS) is potentially inefficient due to non-dynamic methylation across a large fraction of CpG cites as well as the majority of WGBS reads being non-informative (Ziller et al., 2013). Nevertheless, sequencing costs are decreasing dramatically and more comprehensive DNA methylation datasets would become publicly available once whole-genome bisulfite and oxidative bisulfite sequencing becomes mainstream. Development of more accurate epigenetic biomarkers by relying on whole genome sequencing data will be a hot topic in the next years. Future work based on these data should be even more exciting and would have important implications for human health.

## Author Contributions

All authors were involved in literature review, writing the manuscript, and figure preparation.

## Conflict of Interest

The authors declare that the research was conducted in the absence of any commercial or financial relationships that could be construed as a potential conflict of interest.

## Supplementary Material

TABLE S1 Summary of the potential blood-based epigenetic biomarkers for Alzheimer’s disease, cardiovascular disease, and Type 2 diabetes.
