# Supplementary material for: DNA Methylation Biomarkers in Aging and Age-Related Diseases
Source: Front Genet. 2020 Mar 10;11:171. doi: 10.3389/fgene.2020.00171 (PMC7076122; doi:10.3389/fgene.2020.00171)
Supplement: TABLE S1 — Summary of the potential blood-based epigenetic biomarkers for Alzheimer’s disease, cardiovascular disease, and Type 2 diabetes. [file Table_1.DOCX]

| **Disease** | **Epigenetic modification** | **Potential Biomarker** | **Reference** |
| --- | --- | --- | --- |
| **Alzheimer’s Disease** | **DNA Methylation** | *TREM2* | (Ozaki et al., 2017) |
|  |  | *PICALM* | (Mercorio et al., 2018) |
|  |  | *BDNF* | (Xie et al., 2017) |
|  |  | *SPINT1* | (Mano et al., 2017) |
|  |  | *COASY* | (Kobayashi et al., 2016) |
|  |  | *NCAPH2/LMF2* | (Shinagawa et al., 2016) |
| **Cardiovascular Disease** | **DNA Methylation** | *LDLR* | (Infante et al., 2019) |
|  |  | *SGK1, SMARC4, and ZFHX3* | (Nakatochi et al., 2017) |
|  |  | *SLC9A1, SLC1A5, and TNRC6C* | (Westerman et al., 2018) |
|  |  | *DTX3L- PARP9 and NLRC5.* | (Akinyemiju et al., 2018) |
|  |  | *TRAF3* | (Gallego-Fabrega et al., 2016b) |
|  |  | *PPM1A* | (Gallego-Fabrega et al., 2016a) |
| **Type 2 Diabetes** | **DNA Methylation** | *PPAR gamma* | (van Otterdijk et al., 2017) |
|  |  | *PDK4* | (van Otterdijk et al., 2017) |
|  |  | *FTO* | (van Otterdijk et al., 2017), (Toperoff et al., 2012) |
|  |  | *TCF7L2* | (Canivell et al., 2014) |
|  |  | *GCK* | (Tang et al., 2014b) |
|  |  | *PRKCZ* | (Zou et al., 2013) |
|  |  | *BCL11A* | (Tang et al., 2014a) |
|  |  | *GIPR* | (Canivell et al., 2013) |
|  |  | *IGFBP-7* | (Gu et al., 2013) |
|  |  | *PTPN1* | (Huang et al., 2017) |
|  |  | *CAMK1D* | (Cheng et al., 2014) |
|  |  | *CRY2* | (Cheng et al., 2014) |
|  |  | *CALM2* | (Cheng et al., 2014) |
|  |  | *TLR2* | (Remely et al., 2014 a) |
|  |  | *TLR4* | (Remely et al., 2014 a) |
|  |  | *FFAR3* | (Remely et al., 2014 b) |
|  |  | *MALT1* | (Yuan et al., 2014) |
|  |  | *GPR61* | (Yuan et al., 2014) |
|  |  | *ABCG1* | (Chambers et al., 2015), (Dayeh et al., 2016), (Akinyemiju et al., 2018) |
|  |  | *PHOSPHO1* | (Chambers et al., 2015), (Dayeh et al., 2016) |
|  |  | *SOCS3* | (Chambers et al., 2015), |
|  |  | *SREBF1* | (Chambers et al., 2015), (Walaszczyk et al., 2018) |
|  |  | *TXNIP* | (Chambers et al., 2015), (Kulkarni et al., 2015), (Florath et al., 2016; Soriano-Tárraga et al., 2016), (Walaszczyk et al., 2018) |
|  |  | *LOXL2* | (Walaszczyk et al., 2018) |
|  |  | *SLC1A5* | (Walaszczyk et al., 2018) |
|  |  | *SAMD12* | (Kulkarni et al., 2015) |

Akinyemiju, T., Do, A. N., Patki, A., Aslibekyan, S., Zhi, D., Hidalgo, B., et al. (2018). Epigenome-wide association study of metabolic syndrome in African-American adults. *Clin. Epigenetics* 10, 1–8. doi:10.1186/s13148-018-0483-2.

Canivell, S., Ruano, E. G., Sisó-Almirall, A., Kostov, B., González-de Paz, L., Fernandez-Rebollo, E., et al. (2013). Gastric Inhibitory Polypeptide Receptor Methylation in Newly Diagnosed, Drug-Naïve Patients with Type 2 Diabetes: A Case-Control Study. *PLoS ONE* 8. doi:10.1371/journal.pone.0075474.

Canivell, S., Ruano, E. G., Sisó-Almirall, A., Kostov, B., González-de Paz, L., Fernandez-Rebollo, E., et al. (2014). Differential Methylation of TCF7L2 Promoter in Peripheral Blood DNA in Newly Diagnosed, Drug-Naïve Patients with Type 2 Diabetes. *PLOS ONE* 9, e99310.

Chambers, J. C., Loh, M., Lehne, B., Drong, A., Kriebel, J., Motta, V., et al. (2015). Epigenome-wide association of DNA methylation markers in peripheral blood from Indian Asians and Europeans with incident type 2 diabetes: a nested case-control study. *Lancet Diabetes Endocrinol.* 3, 526–534. doi:10.1016/S2213-8587(15)00127-8.

Cheng, J., Tang, L., Hong, Q., Ye, H., Xu, X., Xu, L., et al. (2014). Investigation into the promoter dna methylation of three genes (CAMK1D, CRY2 and CALM2) in the peripheral blood of patients with type 2 diabetes. *Exp. Ther. Med.* 8, 579–584. doi:10.3892/etm.2014.1766.

Dayeh, T., Tuomi, T., Almgren, P., Perfilyev, A., Jansson, P.-A., de Mello, V. D., et al. (2016). DNA methylation of loci within *ABCG1* and *PHOSPHO1* in blood DNA is associated with future type 2 diabetes risk. *Epigenetics* 11, 482–488. doi:10.1080/15592294.2016.1178418.

Florath, I., Butterbach, K., Heiss, J., Bewerunge-Hudler, M., Zhang, Y., Schöttker, B., et al. (2016). Type 2 diabetes and leucocyte DNA methylation: an epigenome-wide association study in over 1,500 older adults. *Diabetologia* 59, 130–138. doi:10.1007/s00125-015-3773-7.

Gallego-Fabrega, C., Carrera, C., Reny, J.-L., Fontana, P., Slowik, A., Pera, J., et al. (2016a). *PPM1A* Methylation Is Associated With Vascular Recurrence in Aspirin-Treated Patients. *Stroke* 47, 1926–1929. doi:10.1161/STROKEAHA.116.013340.

Gallego-Fabrega, C., Carrera, C., Reny, J.-L., Fontana, P., Slowik, A., Pera, J., et al. (2016b). *TRAF3* Epigenetic Regulation Is Associated With Vascular Recurrence in Patients With Ischemic Stroke. *Stroke* 47, 1180–1186. doi:10.1161/STROKEAHA.115.012237.

Gu, H. F., Gu, T., Hilding, A., Zhu, Y., Kärvestedt, L., Östenson, C. G., et al. (2013). Evaluation of IGFBP-7 DNA methylation changes and serum protein variation in Swedish subjects with and without type 2 diabetes. *Clin. Epigenetics* 5, 1–7. doi:10.1186/1868-7083-5-20.

Huang, Q., Han, L., Liu, Y., Wang, C., Duan, D., Lu, N., et al. (2017). Elevation of PTPN1 promoter methylation is a significant risk factor of type 2 diabetes in the Chinese population. *Exp. Ther. Med.* 14, 2976–2982. doi:10.3892/etm.2017.4924.

Infante, T., Forte, E., Schiano, C., Punzo, B., Cademartiri, F., Cavaliere, C., et al. (2019). Evidence of association of circulating epigenetic-sensitive biomarkers with suspected coronary heart disease evaluated by Cardiac Computed Tomography. *PLOS ONE* 14, e0210909. doi:10.1371/journal.pone.0210909.

Kobayashi, N., Shinagawa, S., Nagata, T., Shimada, K., Shibata, N., Ohnuma, T., et al. (2016). Usefulness of DNA Methylation Levels in COASY and SPINT1 Gene Promoter Regions as Biomarkers in Diagnosis of Alzheimer’s Disease and Amnestic Mild Cognitive Impairment. *PLOS ONE* 11, e0168816. doi:10.1371/journal.pone.0168816.

Kulkarni, H., Kos, M. Z., Neary, J., Dyer, T. D., Kent, J. W., Göring, H. H. H., et al. (2015a). Novel epigenetic determinants of type 2 diabetes in Mexican-American families. *Hum. Mol. Genet.* 24, 5330–5344. doi:10.1093/hmg/ddv232.

Kulkarni, H., Kos, M. Z., Neary, J., Dyer, T. D., Kent, J. W., Göring, H. H. H., et al. (2015b). Novel epigenetic determinants of type 2 diabetes in Mexican-American families. *Hum. Mol. Genet.* 24, 5330–5344. doi:10.1093/hmg/ddv232.

Mano, T., Nagata, K., Nonaka, T., Tarutani, A., Imamura, T., Hashimoto, T., et al. (2017). Neuron-specific methylome analysis reveals epigenetic regulation and tau-related dysfunction of BRCA1 in Alzheimer’s disease. *Proc. Natl. Acad. Sci.* 114, E9645–E9654. doi:10.1073/pnas.1707151114.

Mercorio, R., Pergoli, L., Galimberti, D., Favero, C., Carugno, M., Dalla Valle, E., et al. (2018). PICALM Gene Methylation in Blood of Alzheimer’s Disease Patients Is Associated with Cognitive Decline. *J. Alzheimers Dis.* 65, 283–292. doi:10.3233/JAD-180242.

Nakatochi, M., Ichihara, S., Yamamoto, K., Naruse, K., Yokota, S., Asano, H., et al. (2017). Epigenome-wide association of myocardial infarction with DNA methylation sites at loci related to cardiovascular disease. *Clin. Epigenetics* 9, 54. doi:10.1186/s13148-017-0353-3.

Ozaki, Y., Yoshino, Y., Yamazaki, K., Sao, T., Mori, Y., Ochi, S., et al. (2017). DNA methylation changes at TREM2 intron 1 and TREM2 mRNA expression in patients with Alzheimer’s disease. *J. Psychiatr. Res.* 92, 74–80. doi:10.1016/j.jpsychires.2017.04.003.

Remely, M., Aumueller, E., Jahn, D., Hippe, B., Brath, H., and Haslberger, A. G. (2014). Microbiota and epigenetic regulation of inflammatory mediators in type 2 diabetes and obesity. *Benef. Microbes* 5, 33–43. doi:10.3920/BM2013.006.

Shinagawa, S., Kobayashi, N., Nagata, T., Kusaka, A., Yamada, H., Kondo, K., et al. (2016). DNA methylation in the NCAPH2/LMF2 promoter region is associated with hippocampal atrophy in Alzheimer’s disease and amnesic mild cognitive impairment patients. *Neurosci. Lett.* 629, 33–37. doi:10.1016/j.neulet.2016.06.055.

Soriano-Tárraga, C., Jiménez-Conde, J., Giralt-Steinhauer, E., Mola-Caminal, M., Vivanco-Hidalgo, R. M., Ois, A., et al. (2016). Epigenome-wide association study identifies *TXNIP* gene associated with type 2 diabetes mellitus and sustained hyperglycemia. *Hum. Mol. Genet.* 25, 609–619. doi:10.1093/hmg/ddv493.

Tang, L., Wang, L., Ye, H., Xu, X., Hong, Q., Wang, H., et al. (2014a). BCL11A gene DNA methylation contributes to the risk of type 2 diabetes in males. *Exp. Ther. Med.* 8, 459–463. doi:10.3892/etm.2014.1783.

Tang, L., Ye, H., Hong, Q., Wang, L., Wang, Q., Wang, H., et al. (2014b). Elevated CpG island methylation of GCK gene predicts the risk of type 2 diabetes in Chinese males. *Gene* 547, 329–333. doi:10.1016/j.gene.2014.06.062.

Toperoff, G., Aran, D., Kark, J. D., Rosenberg, M., Dubnikov, T., Nissan, B., et al. (2012). Genome-wide survey reveals predisposing diabetes type 2-related DNA methylation variations in human peripheral blood. *Hum. Mol. Genet.* 21, 371–383. doi:10.1093/hmg/ddr472.

van Otterdijk, S. D., Binder, A. M., Szarc vel Szic, K., Schwald, J., and Michels, K. B. (2017). DNA methylation of candidate genes in peripheral blood from patients with type 2 diabetes or the metabolic syndrome. *PLOS ONE* 12, e0180955.

Walaszczyk, E., Luijten, M., Spijkerman, A. M. W., Bonder, M. J., Lutgers, H. L., Snieder, H., et al. (2018). DNA methylation markers associated with type 2 diabetes, fasting glucose and HbA1c levels: a systematic review and replication in a case–control sample of the Lifelines study. *Diabetologia* 61, 354–368. doi:10.1007/s00125-017-4497-7.

Westerman, K., Sebastiani, P., Jacques, P., Liu, S., DeMeo, D., and Ordovás, J. M. (2018). DNA methylation modules associate with incident cardiovascular disease and cumulative risk factor exposure. Genomics doi:10.1101/471722.

Xie, B., Xu, Y., Liu, Z., Liu, W., Jiang, L., Zhang, R., et al. (2017). Elevation of Peripheral BDNF Promoter Methylation Predicts Conversion from Amnestic Mild Cognitive Impairment to Alzheimer’s Disease: A 5-Year Longitudinal Study. *J. Alzheimers Dis.* 56, 391–401. doi:10.3233/JAD-160954.

Yuan, W., Xia, Y., Bell, C. G., Yet, I., Ferreira, T., Ward, K. J., et al. (2014). An integrated epigenomic analysis for type 2 diabetes susceptibility loci in monozygotic twins. *Nat. Commun.* 5. doi:10.1038/ncomms6719.

Zou, L., Yan, S., Guan, X., Pan, Y., and Qu, X. (2013). Hypermethylation of the PRKCZ gene in type 2 diabetes mellitus. *J. Diabetes Res.* 2013. doi:10.1155/2013/721493.
